# How Self-Directed e-Learning Contributes to Training for Medical Licentiate Practitioners in Zambia: Evaluation of the Pilot Phase of a Mixed-Methods Study

**DOI:** 10.2196/10222

**Published:** 2018-11-27

**Authors:** Sandra Barteit, Albrecht Jahn, Annel Bowa, Sigrid Lüders, Gregory Malunga, Clemence Marimo, Sigrid Wolter, Florian Neuhann

**Affiliations:** 1 Heidelberg Institute of Global Health Heidelberg University Heidelberg Germany; 2 Chainama College of Health Sciences Lusaka Zambia; 3 SolidarMed Lusaka Zambia; 4 School of Medicine University of Zambia Lusaka Zambia

**Keywords:** evaluation, medical e-learning, intervention, sustainability, effectiveness, adoption, health care workers, rural health, sub-Saharan Africa

## Abstract

**Background:**

Zambia faces a severe shortage of health workers, particularly in rural areas. To tackle this shortage, the Medical Licentiate program was initiated at Chainama College of Health Sciences in the capital, Lusaka, in 2002. The objective of the program was to alleviate the shortage of human resources in curative care. On-the-job training is conducted in decentralized teaching hospitals throughout Zambia. However, the program faces significant challenges such as shortages of senior medical instructors and learning materials.

**Objective:**

Our aim was to address these challenges by introducing a self-directed, e-learning platform with an offline tablet as part of a collaborative blended-learning intervention to supplement local teaching and training.

**Methods:**

The pilot phase of the e-learning platform was evaluated using a mixed-methods approach with a convergent parallel design. Various methods were employed to test the data’s adequacy and potential for generating valid results. Methods included questionnaires according to the technology acceptance model and information system success model by DeLone and McLean, semistructured interviews, learner diaries, pretesting, the collection of usage data, exam results, demographics, and informal feedback. Outcome measures included usage, adoption, efficiency, acceptance, user-friendliness, and gained knowledge and skills.

**Results:**

In total, 52 students and 17 medical instructors participated in the pilot evaluation. The questionnaire results showed a high acceptance of the technology (>80%) and high agreement (>75%) with the e-learning platform. Semistructured interview results showed an overall appreciation of the e-learning intervention, but the need for more e-learning materials. Respondents identified a need for multimedia materials that transfer skills such as medical procedure visualization and interactive exercises to practice procedural knowledge. The learning diaries identified the lack of specific learning materials and potential shortcomings of existing learning materials. However, students were satisfied with the current e-learning content. The majority of students used the e-learning platform offline on their tablets; online e-learning was underutilized.

**Conclusions:**

The pilot phase of the tablet-based e-learning platform to support the self-directed learning intervention was well received and appreciated by students and medical instructors of Chainama College of Health Sciences. E-learning for knowledge acquisition appears to be adequate and feasible for this low-resource educational environment. Our evaluation results guide the further development of the full implementation of the e-learning platform in this educational setting. E-learning materials should reflect curriculum requirements, and additional multimedia and interactive content is needed, as well as improved integration and active participation from medical instructors in the e-learning processes.

## Introduction

Zambia’s severe lack of health workers is leaving basic population health needs unmet [1], which is further aggravated by an overabundance of medical doctors in urban areas [2]. Consequently, rural health clinics are often understaffed and managed by health workers who lack the medical qualifications to manage their patient population adequately [2,3]. A human resource upscale of 140% is necessary to reduce the current deficiency, which may be further intensified by population growth that may double the health worker shortage by 2035 [2]. More health worker training is needed “with measures to mitigate attrition and to increase productivity” [4]. As part of Zambia’s national response to the health worker deficiency, especially in rural areas, the Medical Licentiate (ML) program was established at Chainama College of Health Sciences (CCHS), in 2002 [5]. Initially, the program was targeted at in-service clinical officers to upgrade their medical skills in order to perform various essential operations and manage Level 1 health care facilities. Graduated MLs are placed primarily in district hospitals on the periphery of Zambia with a focus on internal medicine, pediatrics, surgery, gynecology, and obstetrics. The ML program has proven valuable to date, particularly for high retention [6] and equal distribution of MLs throughout rural Zambia [4,5]. An essential part of the ML training includes clinical rotations, which currently lack adequate onsite senior supervision, mentorship, and learning resources. Medical instructors, the majority of which are physicians, are often faced with a double burden, since their appointment to the ML program is in addition to their daily clinical duties. Furthermore, appointed medical instructors have no didactic training for this program. Learning materials are scarce in the rural training sites, although technologies for medical education are widely available even in low-resource countries [7].

In Africa, the number of households with a personal computer has significantly increased as reported by the International Telecommunications Union [8]. E-learning for medical education offers significant potential for knowledge acquisition [9], particularly for health care training, monitoring, diagnostics, and new analysis methodologies [8]. The advantages of e-learning have been widely discussed [10-13] and could potentially address the bottlenecks faced by low-resource countries scaling up health worker numbers. E-learning is flexible and can provide self-directed, local, and personalized training, and it can easily be scaled to provide access to current educational materials even in the most remote areas.

Thus, Heidelberg Institute of Public Health in partnership with the Swiss nongovernmental organization, SolidarMed, and CCHS implemented a medical e-learning platform to strengthen the ML program. A 2-week fact-finding mission was conducted in November 2015, followed by an e-learning platform piloted from January-July 2016. For cost-effectiveness, we focused on open-source software and low-cost qualitative hardware. Initial e-learning materials included lecture notes taken from a previous e-learning project in Malawi and then adapted to the Zambian context [14]. The content was targeted at ML students only. There was no content specifically for medical instructors as they were instead to make use of the e-learning platform as a teaching method. During the pilot phase, additional e-learning materials were developed and made available per the ML curriculum, including medical books, treatment guidelines and procedures, virtual patients (interactive patient cases), and lecture notes. ML students in their final study year were given tablets preloaded with e-learning content for offline usage (see Multimedia Appendix 1 for a full list of e-learning materials). Furthermore, a local Web server was implemented on campus, and local information and communications technology (ICT) support was trained in e-learning administration and support. The objective of the pilot phase was to test the feasibility of a blended learning approach for this educational environment since blended learning has shown to be effective in strengthening educational interventions in health care in similar settings [9,10]. In this paper, we introduce the pilot evaluation methods in detail, discuss results, reflect on shortcomings, and propose recommendations based on our findings. The adequacy of the mixed method approach we employed was also evaluated as a secondary objective of the pilot phase.

## Methods

### Overview

**Figure 1:**
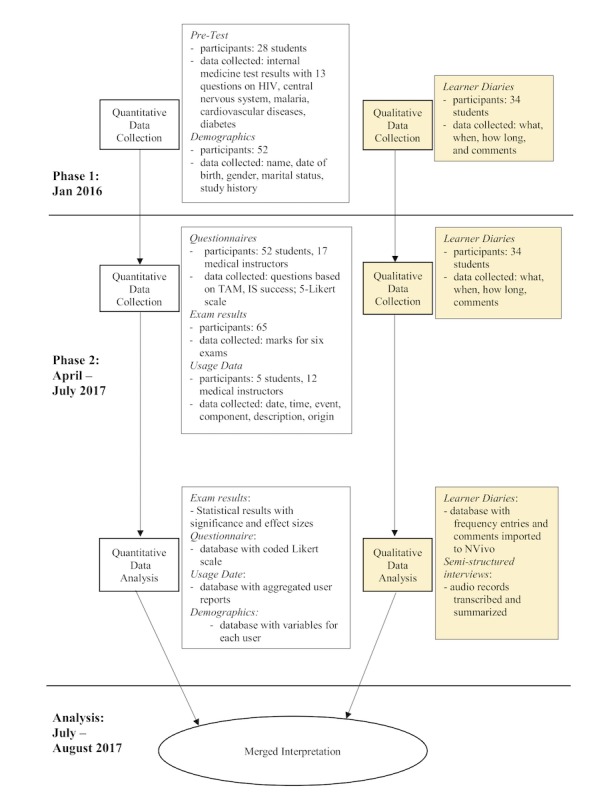
Convergent mixed-methods design of the evaluation of the Medical Licentiate (ML) e-learning intervention. IS: Information System Success Model; TAM: Technology Acceptance Model.

The e-learning evaluation followed a mixed-methods approach by employing a convergent design [12,13] (see Figure 1) to obtain data from various perspectives and gain a comprehensive understanding of the following study questions [12]:

1. How adequate is the employed technology?
2. How do students use the e-learning platform?
3. How does e-learning enable and support learning and teaching?
4. How useful are e-learning materials?
5. What challenges in e-learning are encountered?

Data collection followed a purposive, nonrandom sampling procedure since all CCHS students and instructors involved with the e-learning platform were included. The Consolidated Criteria for Reporting Qualitative Research guided the qualitative data collection [14]. The quantitative data collection followed guidelines for “Assessing Rigour in Quantitative Health Sciences Research” [13]. Results were validated by merging and comparing both sets of data [12].

Qualitative data included (1) learner diaries given to students to provide feedback on e-learning materials and usage, (2) six semistructured student interviews using 12 guiding questions with a purposive selection including 3 females and 3 males, ages ≤30, ≥31-45, >45 years (see Multimedia Appendix 2), and (3) informal feedback from student statements and the status quo of tablets (ie, condition of tablets, installed apps, specific tablet configuration) during general tablet maintenance at the end of the study year in July 2016.

Quantitative data included the following:

1. Knowledge assessment by pretest and posttest (see Multimedia Appendix 3) with multiple-choice and short-answer questions on internal medicine.
2. Two questionnaires (see Multimedia Appendices 4 and 5) using a 5-point Likert scale based on the Technology Acceptance Model (TAM), which assesses students’ acceptance and use of technology based on perceived usefulness and ease-of-use, and the Information System (IS) Success Model [15-17], which assesses the success of the information system’s components [18] based on six interrelated and interdependent dimensions: the student questionnaire with 60 statements (36 from IS and 24 from TAM), and the medical instructors’ questionnaire with 32 statements (15 from IS and 17 from TAM).
3. Usage data of the e-learning platform based on inbuilt Moodle statistics.
4. Demographics of ML students and medical instructors comprising both cohorts’ dates of birth, gender, and years of medical practice.
5. Exam results of ML students’ end-of-year exam.

### Ethics

The Biomedical Research Ethics Committee of the University of Zambia approved this study’s protocol, as well as the ethical committee of the University Hospital Heidelberg, Germany. All participating students agreed before taking part in this study that the user data (frequency of use) would be tracked and analyzed. All approached and selected interview participants were informed about the scope and purpose of the interviews and their right to withdraw. All participants gave written consent. All students and instructors in the study were treated with an ethic of respect. All data gathered in the survey were anonymized before the analyses.

### Data Analysis

#### Quantitative Data

Pretests for knowledge assessment were marked by medical instructors specialized in internal medicine. Questionnaire results were aggregated in MS Excel and analyzed according to ordinal data with the mean as the best measure of central tendency. Usage data were exported in an MS Excel format and analyzed according to the login frequency of individual users. Demographic data were descriptively analyzed in MS Excel with graphs for visualization. Exam results were checked for correlation (one-way analysis of variance) with questionnaire results and correlation with the frequency of participation in learner diaries.

#### Qualitative Data

Contents of the learner diaries were transcribed in MS Excel and analyzed according to word frequency and number of entries. Interviews were transcribed with word-processing software and further analyzed with computer-assisted qualitative data analysis software (NVivo) based on grounded theory. The coding followed the qualitative data analysis process is described in the Coding Manual for Qualitative Researchers [19]. Data were coded by hypothesis coding [19] and conducted in two coding cycles. Informal feedback was sought to complement data and widen insights if deemed necessary by the researchers.

## Results

### Summary

Overall, the pilot phase of the e-learning platform comprised the following results:

1. E-learning server set up locally on CCHS campus.
2. Tablets fit collegiate requirements of ML students.
3. Establishment of ICT support infrastructure for e-learning.
4. ICT training for e-learning administration and support.
5. Support materials created and provided for e-learning usage proved supportive.
6. ML students found the e-learning platform useful for their needs.
7. A general acceptance of e-learning platform by medical instructors.
8. Positive feedback to e-learning platform from students and medical instructors.

Results are presented concisely according to the respective evaluation method and then according to the study questions.

### Demographics

**Figure 2:**
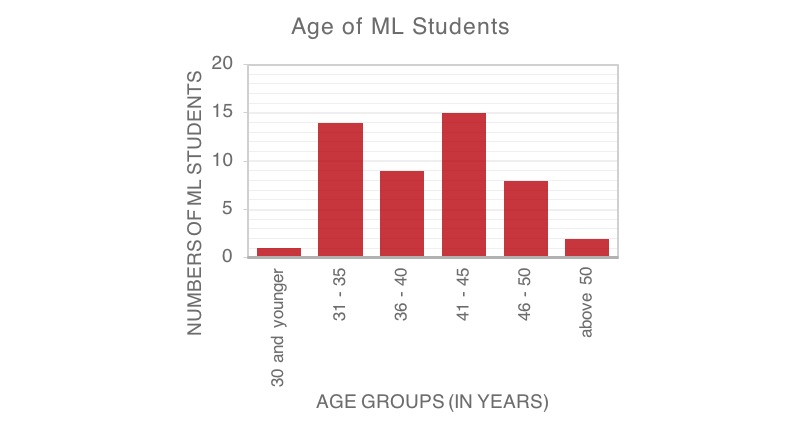
Age of Medical Licentiate (ML) students depicted in six age groups.

**Figure 3:**
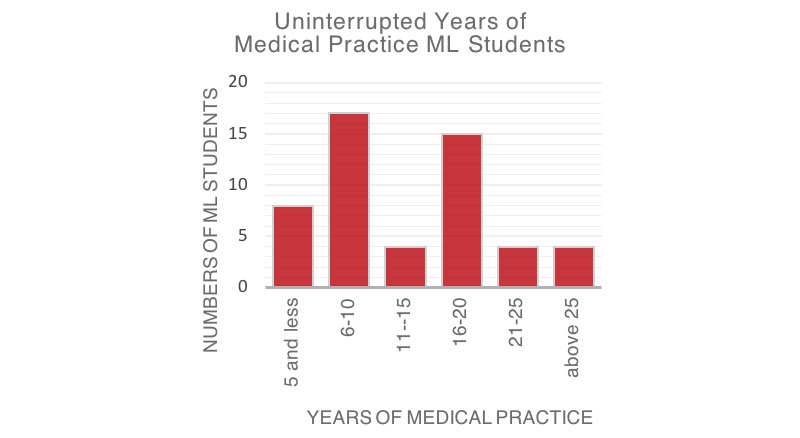
Uninterrupted years of medical practice of Medical Licentiate (ML) students.

In total, 14 females and 38 males (52/65 students) participated in the study, except for the final exam in which all 65 students participated. The ML students are a heterogeneous group with the youngest student aged 27 and the eldest aged 54 years (see Figure 2). Student distribution reflected clinical experience ranging from novice to quite experienced adult learners (see Figure 3): 85% (44/52) had more than 10 years of clinical experience, 8% (4/52) more than 25 years, and 15% (8/52) fewer than 5 years of continuous medical practice (see Figure 3). Medical instructors (2 female, 14 male) were also heterogeneous with ages ranging from 36-56 years. Most of the medical instructors (14/16, 88%) had more than 10 years of experience in professional medical practice.

### Exam Results

**Figure 4:**
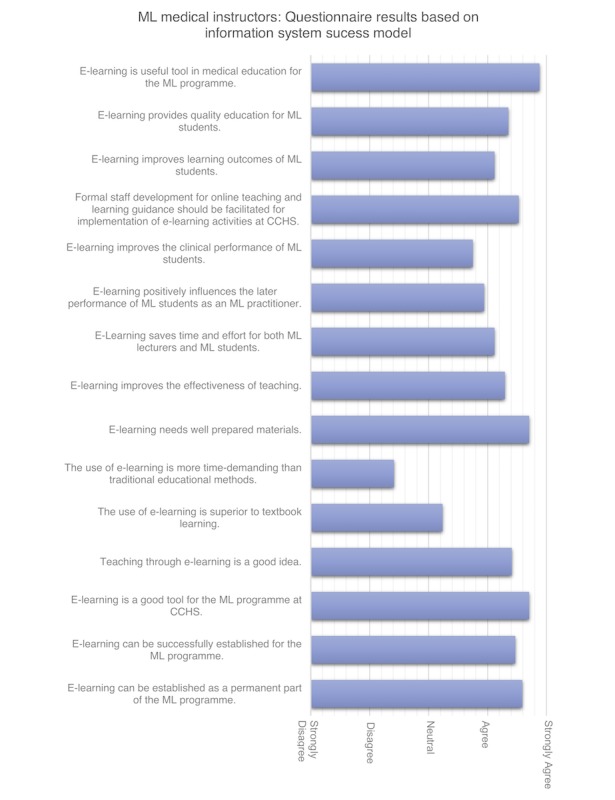
Questionnaire results of medical instructors based on the Information System (IS) Success Model. CCHS: Chainama College of Health Sciences; ML: Medical Licentiate.

**Figure 5:**
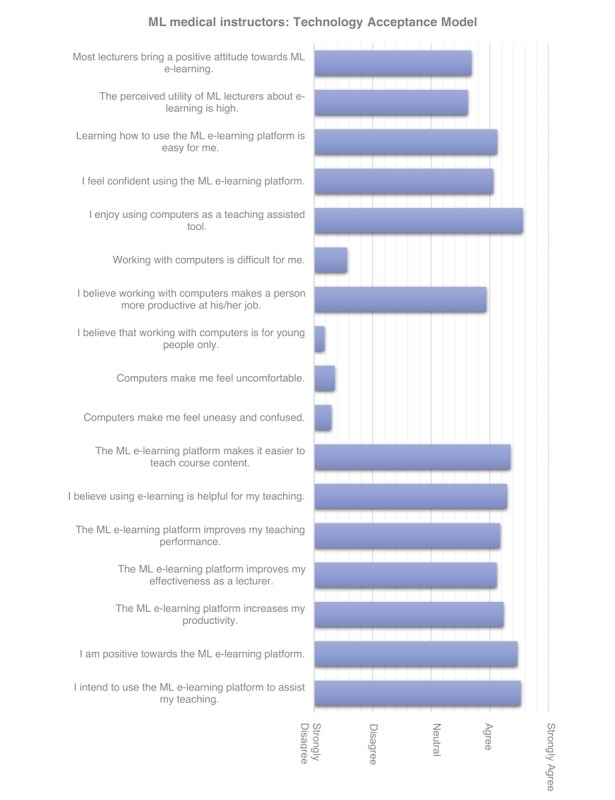
Questionnaire results of medical instructors based on Technology Acceptance Model (TAM). ML: Medical Licentiate.

**Figure 6:**
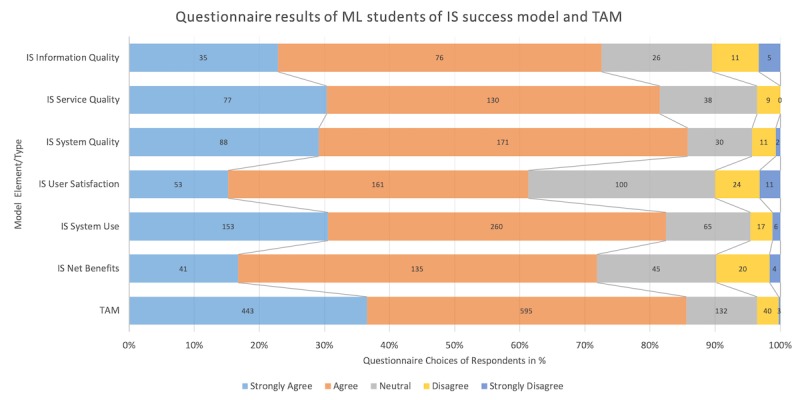
Questionnaire results of Medical Licentiate (ML) students by model type (Information System [IS] Success Model and Technology Acceptance Model [TAM]).

All ML students in their last study year had a final exam (65/65). For all 65 ML students who participated in the exam, results were checked for correlation (one-way analysis of variance) with questionnaire results covering all factors of the IS success model (ie, information quality, service quality, system quality, user satisfaction, system use, net benefits) and TAM. Figures 4 and 5 present the results for medical instructors based on the IS success and TAM models. Figure 6 shows the results for ML students by IS success model and TAM. A correlation was also checked for self-reported usage in the learner diaries. No correlations were found.

### Usage Data

In total, 12 medical instructors and 5 students logged into the online e-learning platform, on five unique dates from January-March 2016. Online usage of the e-learning platform proved too infrequent for inclusion in the mixed-methods analysis and results were discarded.

### Learner Diaries

**Figure 7:**
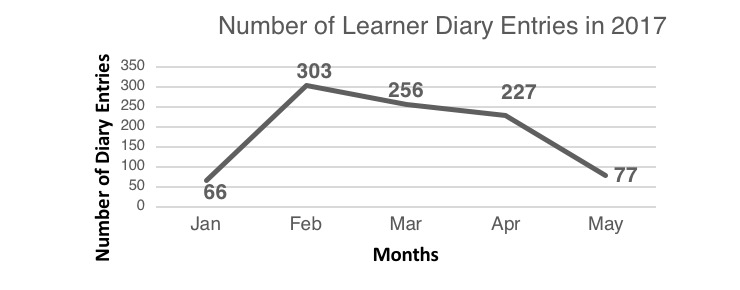
Number of learner diary entries per month during the pilot phase January-May 2017.

Of 52 learner diaries, only 34 were collected (see Multimedia Appendix 6). On average, the diaries contained 27 entries from January-May 2017 (range 2-85 entries) (see Figure 7). The most frequently used comment notes were “good information,” “excellent notes,” “good notes,” “well understood,” “well summarized,” “good,” “excellent,” and “helpful information.” The rating regarding the content was positive overall: “good information,” “excellent notes,” “good notes,” “well understood,” “well summarized,” “good,” “excellent,” and “helpful information.”

### Pretests and Posttests

Students answered almost all the questions on the pretest correctly. Hence, the pretest did not provide a nuanced result useful for assessing differences in knowledge once the course was completed, so the posttest was omitted (see Multimedia Appendix 3 for pretests).

### Questionnaires

In total, 52 students and 16 medical instructors completed the questionnaire (see Figure 6). The correlation was tested for the following variables for both groups: age, gender, and years of medical practice. We found that the older the students were, the higher their satisfaction with the e-learning intervention (*P*=.02). Male students were more satisfied with information quality (*P*=.03), and showed higher user satisfaction (*P*=.01), system use (*P*<.001), and technology acceptance (*P*<.001). Years of medical practice did not correlate with questionnaire results.

### Student Interviews

In total, 6 semistructured interviews were held in a secluded office on the CCHS campus in Lusaka. The interview results are included in the following merged interpretation of the results.

### Adequacy of Employed Technology

Overall, the ML e-learning platform was received as beneficial by students, supporting their clinical performance and fitting in well with their clinical duties: “very informative…as when on call, as [the tablet] is easy to carry during ward rounds,” “was reading during evening call in labor ward,” and “reference during ward rounds, was asked a question by consultant – had to make reference, was very helpful.” There was general agreement that studying through e-learning is a good idea and fits well with the learning style of ML students. A student mentioned, “I don’t have to go home to read textbooks” and another said the tablet “is handy” and useful “for consultation.”

The acceptance of e-learning as a new learning technology was high (over 80% of respondents, both ML students and medical instructors). Students were slightly less positive about the information system (over 75% of respondents were positive). Student feedback also confirmed this since they perceived the tablets and e-learning platform as a positive development in the ML program, and the e-learning platform was a status symbol characterizing program development. From both ML students and medical instructors, the questionnaire received the lowest ratings in the categories of information quality, user satisfaction, and net benefits. Students highly rated user satisfaction and net benefits. For most students, using and accessing materials on the tablet was relatively easy. Students stated that the effort to learn how to use the e-learning platform was acceptable, and they perceived it as user-friendly with a suitable user-interface and having appealing features. Students commented that it was “convenient” and “very good to access information.” Most students thought their fellow students brought a positive attitude towards the ML e-learning platform. ML students and medical instructors rated perceived utility as adequate, but the scope of the ML e-learning was regarded as inadequate.

Most medical instructors stated that they enjoyed using computers to assist with teaching and disagreed that computers made them feel uncomfortable, uneasy, or confused. Medical instructors agreed that computers were not only for young people. The majority of medical instructors intended to use the e-learning platform to assist their teaching. They were positive about the e-learning platform and considered it to be helpful by increasing their productivity and enhancing their teaching performance. Learning how to use the ML e-learning platform was easy for most medical instructors, as one mentioned that it was “not an effort.” Confidence about using the e-learning platform was rated as reasonable by most medical instructors. Computers were not perceived to increase job productivity for teaching nor daily medical practice. Medical instructors using e-learning rated their positive attitude as higher towards the e-learning platform than they rated their medical instructor colleagues.

### Usage of e-Learning Platform

Most students reported having accessed the platform frequently, usually on a daily basis. A few students reported difficulties using the platform because of technical problems. One student mentioned having “had little opportunities to effectively use the facility since faulty settings prevented downloading of contents onto the tablet.” A few students indicated that they did not use the e-learning platform frequently during their practical rotations since they had not found adequate materials for the practical rotations yet. Two interviewees reported that they used the tablet less during electives since subjects such as anesthesia were not yet available on the ML e-learning platform. In the student questionnaires, dependency on the ML e-learning platform was rated low for ML studies and medical practice. Tablet maintenance at the end of the study year showed that many students had downloaded additional apps such as Medscape, an offline handbook for pediatrics, and an offline dictionary for diseases.

Medical instructors evaluated the perceived utility of the online e-learning platform as neutral. The usage data of the ML e-learning platform showed a low involvement. The 12 medical instructors signed in on the online e-learning platform for less than 30 minutes on five dates across January, February, and March 2016.

### Usefulness of Learning Materials

Questionnaire results showed that the ML e-learning platform provided relevant information for ML medical practice. However, the quantity and quality of learning materials were rated as inadequate. One student stated, “books clear, not PowerPoint.” Another stated that “the number of textbooks incorporated was too low for the course” and that “other course contents from the learning curriculum are missing.” Students preferred to have more multimedia-supported content such as “more videos on procedures,” for example, surgery, obstetrics, and gynecology. Available presentations recorded with audio were perceived as “nice videos, well elaborated.” Interviewees requested a quick reference guide or manual, for example, for malnutrition in pediatrics or emergency treatment. Also, it was perceived by ML students to be helpful if more exam-relevant materials were available. Suggestions were made to improve tablet navigation for some e-learning content.

Medical instructors disclosed a high level of agreement that e-learning is a useful and valuable tool that could be established as a core component of the ML program. E-learning was not perceived by medical instructors to be more time-demanding than traditional teaching and learning methods.

### Enabling Medical Learning With the e-Learning Platform

Students perceived the e-learning platform as supportive for improving their medical performance. They found it useful for their studies and fostered clinical thinking. The enhancement of learning performance was rated as satisfactory, and the net benefits as good since e-learning saved students time when they searched for relevant learning materials. E-learning materials complemented face-to-face teaching sessions and led to group discussions. An ML student reported having “found new information from previous notes given in class, very educative, had group discussion within my group and compared.” One interviewee suggested students share learning materials on the e-learning platform among each other. Some students requested a discussion board with medical instructors giving feedback to students’ questions and comments.

Medical instructors reported that they had a positive attitude towards the ML e-learning platform to provide quality education leading to improved learning outcomes for students. However, this agreement was lower for e-learning positively influencing a student’s performance as an ML practitioner. Medical instructors were mostly neutral about e-learning being better than textbook learning, whereas there was a general agreement that e-learning could improve teaching effectiveness.

### Challenges With the e-Learning Platform

Students found the lack of a SIM card slot in the tablet as a limiting factor: “if it [tablet] has a SIM card,” “need for tablet with SIM card provision for Internet connectivity,” and “it would be more wonderful if the tablet had provisions to put a SIM card for network rather than depending on WiFi”. Mobile data was perceived as a crucial feature, especially in rural practical sites with limited Internet access: “with challenges in accessing Internet, the tablets have no provision for SIM card and there is no Internet in school,” “only that limited places have Internet services,” “no access to WiFi,” “Internet was off,” and “no WiFi.”

Another challenge was the level of technical support for the e-learning platform and the tablets, which students perceived as insufficient. Students reported that support has “not helped” and at times there was “no feedback from IT.”

In general, access and usage of the online e-learning platform received the lowest ratings from students. A few students rated their skills as inadequate to make full use of the ML e-learning platform and said it, “needs orientation and practice” and that at times they “needed to consult someone,” since currently there is “no coaching.” Provided tutorials do support students, but additional and more detailed materials were requested. Questionnaire results underlined the need for e-learning training and the need for formal staff development. Medical instructors mentioned “some [medical lecturers] need orientation,” and another admitted “I’ve had a few challenges.”

## Discussion

### Principal Results

The pilot phase of the e-learning platform continued over 6 months and proved that the e-learning platform and the offline tablet-based component are feasible tools for teaching and learning within this low-resource environment.

Initially, only a small number of interactive and multimedia learning materials were available on the e-learning platform. Most of the e-learning materials consisted of lecture notes that were primarily used as presentations within a class and not intended as comprehensive e-learning materials. Thus, these materials were not ideal learning materials for students. The rating of the category of information quality was low, possibly due to the predominance of lecture notes on the e-learning platform. User satisfaction and net benefits were also rated comparatively low, showing that the e-learning platform was not sufficiently meeting the students’ needs. Increasing the quantity and quality of e-learning materials and adapting available materials might address these shortcomings. The demand for more e-learning materials was reflected in the usage of various other medical tablet apps, such as Medscape and medical dictionaries downloaded on the tablets. Furthermore, students felt that the tablet was not as useful as it could be since it did not have a SIM card slot that would allow for mobile data usage, and there was a need for comprehensive training. A SIM card would enable students to access online materials even in rural areas of Zambia. When we decided on tablets for the pilot phase, we seriously considered associated costs, for example, students paying for the tablet. Therefore, we selected a comparatively inexpensive device that did not provide a SIM card slot. Although usage and materials of the online e-learning platform show a clear need for improvement, ML students regarded the tablets and the e-learning platform as a privilege and a development in the ML program.

Older students were more satisfied with the e-learning intervention than younger students. It is possible that they had not been exposed to as much technology throughout their lives as the younger students and thus perceived it as extraordinary to include this technology in their educational environment.

Medical instructors perceived the e-learning platform as quite favorable for the ML program. However, the intended high use of the online e-learning platform was not met by the actual usage, which was low, since only a few medical instructors logged in. Similar findings were shared in another study in India [20], in which e-learning users reported positive feedback, but utilization was low. Some medical instructors in this study stated that they had never used the platform. Therefore, the evaluation of medical instructor use can show only general trends. Involving medical instructors more actively in e-learning is a significant challenge since medical instructors are scarce even within the ML program. A substantial investment by medical instructors and the college is necessary to develop more e-learning materials. A potential enabling factor could be making e-learning more useful to medical instructors and increasing the effectiveness of the e-learning platform, such as introducing an electronic student evaluation.

The evaluation methods were part of the pilot. The mixed method approach was chosen to provide a more comprehensive understanding of the evaluation results [21,22]. To our knowledge, this is one of the first studies to provide a self-directed, offline e-learning approach for a low-resource setting evaluated with a mixed-methods approach. Most e-learning interventions provide an online setup accessed with personal computers. However, our search did yield publications that reported offline approaches with personal computers in low- and lower-middle-income settings [23-26]. The reported methods resulted in varying levels of insights and also unveiled methods that needed adaption to yield evaluation results, such as the knowledge assessment with the student pretest that proved too easy, thus producing no knowledge nuances. The online usage data showed to be insufficient since only a few students and medical instructors logged in on the online platform. The e-learning platform was predominantly accessed offline. Thus, online data were scarce. Lessons learned from the pretests, and the usage data for the next evaluation phase include increasing the level of difficulty of the knowledge assessment pretest and collecting the usage data of the offline Moodle app on the tablet.

The learner diaries did produce helpful feedback on the usage, quality, and usefulness of learning materials. This finding underlines the students’ perception that the e-learning platform was a valuable improvement. However, their perception of the quality of the learning materials was not revealed. It is possible that learner diaries might offer further insight if selection criteria were structured, that is, including the time of access, the type and specialty of e-learning materials, learning duration, and a scaled rating. However, only a few students filled their learner diary diligently, and some diaries were lost. Overall, the pilot phase of the evaluation methods and results were able to highlight needs and point out further improvement needed for the e-learning platform. Although the results were not able to quantitatively provide insights into knowledge and skills improvement, the e-learning intervention was received as quite positive and may be a factor that increases attraction to the ML program.

### Conclusion

The result of the pilot phase of this e-learning intervention confirms its feasibility within this low-resource environment. Evaluation findings show that the technological framework was useful and supportive for students and was well received and accepted by students and medical instructors. The applied mixed method approach to evaluating this e-learning intervention proved adequate to produce valuable results about the quality of teaching materials, usefulness, usage of the e-learning platform, adequacy of technology and challenges, although, methods like the pre-posttest, learner diaries, and usage data need adaptation to capture meaningful data.

Overall, the e-learning platform has the potential to strengthen ML training in the long-term. However, it is crucial to have a reliable IT infrastructure in place and committed stakeholders, especially engaged medical instructors, to integrate medical education e-learning sustainably and comprehensively in low-resource settings.

## Appendix

Multimedia Appendix 1 Available e-learning contents during pilot phase

Multimedia Appendix 2 Semistructured interviews: Guiding questions.

Multimedia Appendix 3 Knowledge assessment: Pretest.

Multimedia Appendix 4 Questionnaire for students.

Multimedia Appendix 5 Questionnaire for medical instructors.

Multimedia Appendix 6 Exemplary diary entry of Medical Licentiate (ML) student.

## Abbreviations

CCHS: Chainama College of Health Sciences
ICT: information and communications technology
IS: Information System Success Model
ML: Medical Licentiate
TAM: Technology Acceptance Model
