# Supplementary material for: How Self-Directed e-Learning Contributes to Training for Medical Licentiate Practitioners in Zambia: Evaluation of the Pilot Phase of a Mixed-Methods Study
Source: JMIR Med Educ. 2018 Nov 27;4(2):e10222. doi: 10.2196/10222 (PMC6290268; doi:10.2196/10222)
Supplement: Multimedia Appendix 1 [file mededu_v4i2e10222_app1.pdf]

Multimedia Appendix 1. Available e-learning contents during pilot phase

**Internal Medicine**		
Type of Material	Number of Materials	Title of Material
Treatment Guidelines	25 guidelines	Physical Examination, Physical Examination – History Questions, Renal Failure, Blood Culture Diagnostic, Chest X-Ray, Fever, Infections, Pneumonia, Sepsis, Sepsis "The Febrile Patient", Liver Cirrhosis, Cardiac Failure, Hypertension, Deep Vein Thrombosis, Seizures, Stroke, Stroke Protocol, Asthma, OPP/Carbamate Poisoning, OPP/Carbamate Poisoning Protocol, Cryptococcal Meningitis, Anemia in HIV Infection, Tuberculosis, Diabetes Mellitus DKA/HHNS, Diabetes Mellitus
Virtual Patients	3 Virtual Patients	"An offensive smell", "Patient Cases: HIV", "Paralyzed Patient"
Lecture Notes	90 lectures	Angina Pectoris, Acute Coronary Syndromes, Cardiomyopathy, Clinical Examination of the Cardiovascular System, Consequences of poorly controlled hypertension, Glomerulonephritides, Heart Failure, Heart Failure - Case Presentation, Heart Failure - Video Presentation, Heart Failure Pocket Guide, Hypertension and Heart Failure, Hypertension, Hypothyroidism, Infective Endocarditis, Major Manifestations of Cardiovascular Diseases, Management of Patients with Valvular Heart Disease, Pulmonary Arterial Hypertension, Rheumatic Fever, Treatment of Hypertension, Valvular Heart Diseases, Epilepsy, Meningitis, Introduction to Chest Radiology, Peripheral Blood Examination, Adrenal Diseases, Diabetes Mellitus, Diabetes Mellitus for MLS, Diabetic Emergencies, Hyperthyroidism, Hypothyroidism for MLS, Peptic Ulcer, Acute Renal Failure, Acute Kidney Injury and Chronic Kidney Disease, Acute Tubular Necrosis, Chronic Renal Failure, Fluids, Electrolytes and Acid-base Abnormalities, Genitourinary Physiology, Glomerulonephritides, Tubulointerstitial Disease, Nephrotic/Nephritic Syndrome, Common Manifestations of GIT Diseases, Chronic Liver Diseases, Diseases of the Liver, Gastritis, Hepatic Encephalopathy, Hepatitis, Major Manifestations of GIT diseases, Pancreatitis, Peptic Ulcer, Peptic Ulcer Disease, Typhoid Fever, Anaemia, Approach to a Patient with Haematological Disorder, Bleeding Disorders, Leukaemias, Myeloproliferative Disorders, Central Nervous System Infections, Hookworms - Endoscopy Video 1, Hookworms - Endoscopy Video 2, Lung Infection, Salmonellae and Typhoid Fever, Urinary Tract Infections, Diabetes Mellitus – DKA, Poisoning, Poisoning - Video Presentation, Paraneoplastic Syndromes, Thyroid Gland, ART Protocols May 2007, ART Treatment: HIV as a Chronic Disease, ART in Africa, HAART and Renal Dysfunction, Yellow Eyes from the Symptom to a Clinical Diagnosis, Malaria, WHO Malaria Treatment Guidelines 2010, WHO GUIDELINES ON THE USE OF ANTIRETROVIRAL DRUGS FOR TREATING AND PREVENTING HIV INFECTION, Guidelines for Prevention and Treatment of Opportunistic Infections in HIV-Infected Adults and Adolescents, Meningitis, Approach to MDR-TB, Anatomy and Physiology of the Respiratory System,

		Asthma, Bronchogenic Carcinoma, Chest X-Rays Made Simple, Chronic Obstructive Pulmonary Disease (COPD), COPD and Asthma, Examination of the Respiratory System Major Manifestations, Pleural Effusion, Pnemosis Carinii Pneumonia (PCP), Pulmonary Tuberculosis, Respiratory Infections, Rheumatology
Medical Courses	1 course	Abdominal Ultrasound (Abdominal Ultrasound - Introduction; Bileducts, Pancreas; Kidneys: Anatomy; Liver Spleen; Retroperitoneum, Vessels, Lymphatic Nodes; Sonography of the Pleura; Schistosomiasis, Gallbladder and Spleen; The Focused Abdominal Sonography for Trauma Scan (FAST); Upper, Lower, Diffuse Abdominal Pain)
Medical Books	8 books	Atlas of Clinical Diagnosis, Bates' Guide to Physical Examination and History-Taking, Handbook of Clinical and Laboratory Investigation, Harrison - Principles of Internal Medicine 16th Edition, Kumar and Clark's Clinical Medicine 6e, MacLeod's Clinical Examination File, ABC of Dermatology, Neurology in Africa
**Obstetrics and Gynaecology**		
Type of Material	Number of Materials	Title of Material
Lecture Notes	46 lectures	Introduction and Terminology in Gynaecology, Diagnosis of Pregnancy and Physiological Changes in Pregnancy, Focussed Antenatal Care, IUGR and IUFD, Problems in Early Pregnancy: Abortion/Miscarriage, Rh Isoimmunization, Ectopic Pregnancy, Fertilization: Early Embryonic Development, Multiple Pregnancy, Conditions of the Body of the Uterus, Female Reproductive Anatomy, The Female Bony Pelvis and Fetal Skull, Anti-Cancer Drugs, Cervical Cancer Screening and Cancer of the Cervix, Ovarian Cancer, Vulvo-Vaginal Tumors, Gestational Trophoblastic Disease, Infections - Vulvovaginal, Cervical, Endometrial, STDs and PID, Obstetrics protocols and guidelines, WHO Partograph for Beginner, Fetal Monitoring during Labor, Injuries to the Birth Canal, Pain in Labour and Relief, Malpresentation, Theories of Onset of Labour and Normal Labour, Antepartum Fetal Monitoring, Antepartum Hemorrhage, Cardiac Disease in Pregnancy, Diabetes in Pregnancy, HIV in Pregnancy, Hypertensive Disorders in Pregnancy, Malaria in Pregnancy, Nausea and Vomiting in Pregnancy, Neonatal Resuscitation, Polyhydramnios, Oligohydramnios and Prom, Puerperium, Infertility, Gynaecological Procedures: Abortions, Operative Vaginal Delivery, Renal Disease in Pregnancy, Contraception, Epidemiology of Obstetrics
Medical Books	1 book	Dr Michael Breen's Practical Handbook Obstetrics and Gynaecology
**Surgery**		
Type of Material	Number of Materials	Title of Material
Lecture Notes	34 lectures	Acute Abdomen, Benign Enlargement of Prostate, Biopsy, Blood Transfusion, Burns, Carcinoma Breast, Cataract, Childhood Malignancy-Clinical Assessment, Diseases of the Breast,

		Empyema Thoracis-Peumothorax-Haemothorax, Fracture Healing,Methods of Fracture Rx: their Indications and Complications, Glaucoma, Head and Neck Tumours, Hernia, Hydrocele, Hypothyroidism MLs Lecture, Injuries around the Elbow Joint, Musculoskeletal Infections in Children, Non-operative Management of Lower Extremity Fractures in the Adult, Orthopaedic Infections, OSCE Stations, Pancreatic Diseases, Pathophysiology and Principles of Fracture Management in Children, Peritonitis, Presentation Multiple Myeloma, Principles of Surgical Practice, Small Bowel Obstruction, Surgery: History – Preparation – Examination, The Operating Theatre, Thyroid Diseases, Thyroidectomy, Upper Extremity Fractures in Adults, Urology
Medical Books	1 book	Primary Surgery - Volume One: Non-trauma, Primary Surgery - Volume Two: Trauma
**Paediatrics**		
Type of Material	Number of Materials	Title of Material
Lecture Notes	32 lectures	Acute Leukaemias, Childhood Malignancy - Clinical Assessment, Clinical Approach To Anaemia, Clinical Approach to Bleeding, Clinical Approach to Lymphadenopathy, Common Infections, Opportunistic Infections and Other Common Clinical Conditions, Common Paediatric Conditions, Congenital Abnormalities, Congenital Heart Diseases, Diabetes Mellitu, Diarrhoea, Disorders of Platelets, Expanded Programme on Immunization, Failure to Thrive, Haematopoiesis, Haemoglobinopathies, History Taking, HIV - Module 1: Overview of Paediatric HIV, HIV - Module 2: Diagnosis & Staging of Pediatric HIV/AIDS, HIV - Module 3: Antiretroviral Drugs, Malnutrition, Measles, Meningitis, Neonatal Jaundice, Opportunistic Infections, Prematurity and Small for Gestation Age, Rheumatic Fever and RHD, Sickle Cell Anaemia - Acute Complications, Sickle Cell Disease, Water and Electrolyte Disturbances, Whooping Cough
Medical Pictures	31 pictures	Anocutaneous fistula in male, At nephrectomy, Bilateral hernia, Burkitts lymphoma, Cleft palate, Distal penile hypospadias, Double bubble in duodenal atresia, Exposed shunt, FB Lt main bronchus, Frontal ethimoidal encephalocele with cleft lip and palate, Gastroschisis, Healing omphalocele, Hermaphroditism, Hermaphroditism 1, Hirshsprung's disease, Hydrocephalus with subcutaneous shunt, Imperforate anus, Imperforate Anus in female, Imperforate anus post op, Intussusception at operation, Intussusception operation, Intussusceptum at anus, Jejunal atresia, Lt lung collapse due to FB, Perforation of ileum following ileal atresia, Proximal fistula in TOF, Renal mass, Repaired hypospadias, Scrotal hypospadias, Tracheosophageal fistula with gastrostomy-note frothy saliva, Urethral stones 5, Urethral stones
Medical Books	3 books	Child Healthcare - A learning programme for professionals, Pediatric History and Physical Examination, Fourth Edition - E.K. Albright, Manual of Clinical Problems in Pediatrics, Fifth Edition - Roberts
