# Supplementary material for: How Self-Directed e-Learning Contributes to Training for Medical Licentiate Practitioners in Zambia: Evaluation of the Pilot Phase of a Mixed-Methods Study
Source: JMIR Med Educ. 2018 Nov 27;4(2):e10222. doi: 10.2196/10222 (PMC6290268; doi:10.2196/10222)
Supplement: Multimedia Appendix 2 [file mededu_v4i2e10222_app2.pdf]

Multimedia Appendix 2. Semi-structured interviews – Guiding questions

1. Why did you decide to train as a Medical Licentiate? What motivated you to join the ML training program?
2. What do you expect from the ML training? What are your priorities in studying within the ML training program?
3. Has the medical training met your expectations so far? If yes, why? If no, why?
4. What do you enjoy most in your training? Can you give an example? What, if any, positive aspects/experiences have you had regarding the ML training? Why?
5. What do you enjoy least in your training? Can you give an example? What, if any, adverse aspects/experiences have you had regarding the ML training? Why? What is difficult?
6. What hurdles remain to you as a student of the ML training program?
7. How do you get along with the e-learning/mobile learning? How do you wish the ML e-learning/m-learning would support you in regards to the ML training?
8. How do you use the tablet for medical, educational purposes? How frequently? In which ways have you used e-learning and m-learning for your ML training? How have you employed the tablet/e-learning platform for your ML training? Could you describe a typical day/week?
9. How do you wish the lecturers would employ the e-learning/m-learning at CCHS?
10. What do you enjoy most about mobile learning with the ML tablet?
11. What do you enjoy least about mobile learning with the ML tablet?
12. How do you think the e-learning/m-learning could be improved for the ML program?)
