# Supplementary material for: How Self-Directed e-Learning Contributes to Training for Medical Licentiate Practitioners in Zambia: Evaluation of the Pilot Phase of a Mixed-Methods Study
Source: JMIR Med Educ. 2018 Nov 27;4(2):e10222. doi: 10.2196/10222 (PMC6290268; doi:10.2196/10222)
Supplement: Multimedia Appendix 3 [file mededu_v4i2e10222_app3.pdf]

Multimedia Appendix 3. Knowledge Assessment: Pre-Test

1. **CNS**
    a. **List 3 commonest CNS opportunistic infections in HIV.**
    b. **Classify the different types of epilepsy.**
2. **HIV**
    a. **What are the first-line treatment guidelines in Zambia?**
    b. **What do you understand by the term option B+?**
    c. **What are the 3 diagnostics for HIV treatment failure?**
3. **Malaria**
    a. **List 3 common complications of severe Malaria.**
    b. **List 2 presentations of each common complication of severe Malaria.**
    c. **Indicate the treatment guidelines for these complications.**
4. **Cardiovascular diseases**
    a. **Please explain major stroke classifications.**
    b. **What type of stroke is most common?**
    c. **What are risk factors for stroke? List at least 3.**
    d. **What are symptoms for a stroke? List at least 3.**
    e. **List (and explain) important elements for stroke management.**
5. **Diabetes**
    a. **Please explain common clinical features of diabetic foot ulcers.**
    b. **What are the underlying causes of diabetic foot ulcers and what are immediate causes?**
    c. **What is the most common site of a diabetic foot ulcer?**
    d. **List three components of diabetic foot assessments.**
    e. **List 3 complications of diabetic foot ulcer**
    f. **List (and explain) important elements for diabetic ulcer management.**
