# Supplementary material for: How Self-Directed e-Learning Contributes to Training for Medical Licentiate Practitioners in Zambia: Evaluation of the Pilot Phase of a Mixed-Methods Study
Source: JMIR Med Educ. 2018 Nov 27;4(2):e10222. doi: 10.2196/10222 (PMC6290268; doi:10.2196/10222)
Supplement: Multimedia Appendix 4 [file mededu_v4i2e10222_app4.pdf]

Multimedia Appendix 4. Questionnaire Students

1. The ML e-learning provides up-to-date learning materials.
2. Learning materials available on the ML e-learning platform are explicit.
3. The ML e-learning provides information relevant to ML medical practice.
4. The ML e-learning platform provides sufficient learning materials.
5. Through the ML e-learning platform, I get access to learning materials I need in time.
6. The ML e-learning platform provides the proper level of assistance and explanation.
7. The tutorials provided have enhanced my ability to use ML e-learning.
8. The local IT has adequate knowledge to help me if I experience any problems with the ML e-learning
9. The IT provides adequate support to users of the ML e-learning platform.
10. The IT attends to my problems.
11. The ML e-learning platform provides dependable services.
12. The ML e-learning platform provides rapid services.
13. *Perceptions of System Quality*
14. The ML e-learning platform is easy to use.
15. The ML e-learning platform is user-friendly.
16. The ML e-learning platform is easy to learn.
17. The user interface of the ML e-learning is attractive.
18. The ML e-learning platform has attractive features to appeal to users.
19. I could use the ML e-learning platform at any time, anywhere I want.
20. I think that most ML students bring a positive attitude towards the ML e-learning platform.
21. I think that most ML students have a high perceived utility about the ML e-learning platform.
22. I am satisfied with the efficiency of the ML e-learning platform.
23. I will continue to use the ML e-learning platform.
24. Overall, I am very satisfied with the ML e-learning platform.
25. My frequency of using the ML e-learning platform is high.
26. I use the ML e-learning platform daily several times.
27. I depend upon the ML e-learning platform.

28. The ML e-learning platform helps me improve my performance as an ML.
29. The ML e-learning platform helps me think through problems.
30. Using the ML e-learning platform has helped me to accomplish my learning tasks more efficiently.
31. Using the ML e-learning platform has made my learning activities become much easier than without.
32. My learning performance enhanced since using the e-learning platform.
33. I find the ML e-learning platform useful for my studies.
34. The ML e-learning platform saves me money.
35. The e-learning materials improve my clinical performance.
36. I feel that the e-learning platform has a direct positive impact on being an ML practitioner.
37. The ML e-learning platform saves me time.
38. I find the ML e-learning platform easy to use.
39. Learning how to use the ML e-learning platform is easy for me.
40. It is easy to become skilful at using the ML e-learning platform.
41. E-learning improves my learning performance.
42. E-learning makes it easier to study course content.
43. Studying through e-learning is a good idea.
44. Studying through e-learning is a wise idea.
45. I am positive towards e-learning.
46. I intend to be a heavy user of the ML e-learning platform.
47. I feel confident finding information on the ML e-learning platform.
48. I have the necessary skills for using the ML e-learning platform.
49. What e-learning stands for is important for me as an ML student.
50. It is necessary to take e-learning courses to train as an ML.
51. I have no difficulty accessing and using the ML e-learning platform.
52. Using a tablet computer is compatible with all aspects of my studies.
53. I think that using a tablet fits well with the way I like to learn.
54. Using a tablet fits into my learning style.
55. In my job, usage of a tablet and e-learning is important.
56. It is fast to access materials on the tablet.

57. The ML e-learning contents on the tablet load quickly.

58. Learning to operate my tablet is easy for me.

59. My interaction with my tablet is clear and understandable.

60. It is easy for me to become skilful at using my tablet.

61. I find my tablet easy to use.
