# Supplementary material for: How Self-Directed e-Learning Contributes to Training for Medical Licentiate Practitioners in Zambia: Evaluation of the Pilot Phase of a Mixed-Methods Study
Source: JMIR Med Educ. 2018 Nov 27;4(2):e10222. doi: 10.2196/10222 (PMC6290268; doi:10.2196/10222)
Supplement: Multimedia Appendix 5 [file mededu_v4i2e10222_app5.pdf]

Multimedia Appendix 5. Questionnaire Medical Teachers

1. E-learning is a useful tool in medical education.
2. The use of educational technologies serves to provide rich educational resources and experiences.
3. E-learning provides extensive education for learners.
4. E-learning improves participation of students in the educational process.
5. Formal staff development for online teaching and learning guidance should be facilitated for implementation of e-learning activities at CCHS.
6. E-learning improves the effectiveness of teaching.
7. E-Learning saves time and effort for both lecturers and students.
8. E-learning needs well prepared materials.
9. The use of Internet reduces the interest of students for face to face educational activities.
10. E-learning may result in a decline of students performances.
11. E-learning may reduce the interactive discussion between the faculty and students.
12. E-learning may result in difficulty in monitoring and evaluation of the students.
13. The use of e-learning is more time-demanding than traditional educational methods.
14. Technological infrastructure is crucial for successful implementation activities.
15. The use of e-learning is superior to textbook learning.
16. Most lecturers bring a positive attitude towards ML e-learning.
17. The perceived utility of ML lecturers about e-learning is high.
18. Learning how to use the ML e-learning platform is easy for me.
19. I feel confident using the ML e-learning platform.
20. I enjoy using computers as a teaching assisted tool.
21. Working with computers is difficult for me.
22. I believe working with computers makes a person more productive at his/her job.
23. I believe that working with computers is for young people only.
24. Computers make me feel uncomfortable.

25. Computers make me feel uneasy and confused.
26. The ML e-learning platform makes it easier to teach course content.
27. I believe using e-learning is helpful for my teaching.
28. The ML e-learning platform improves my teaching performance.
29. The ML e-learning platform improves my effectiveness as a lecturer.
30. The ML e-learning platform increases my productivity.
31. I am positive towards the ML e-learning platform.
32. I intend to use the ML e-learning platform to assist my teaching.
