# Supplementary material for: How Self-Directed e-Learning Contributes to Training for Medical Licentiate Practitioners in Zambia: Evaluation of the Pilot Phase of a Mixed-Methods Study
Source: JMIR Med Educ. 2018 Nov 27;4(2):e10222. doi: 10.2196/10222 (PMC6290268; doi:10.2196/10222)
Supplement: Multimedia Appendix 6 [file mededu_v4i2e10222_app6.pdf]

Multimedia Appendix 6: Exemplary diary entry of ML student

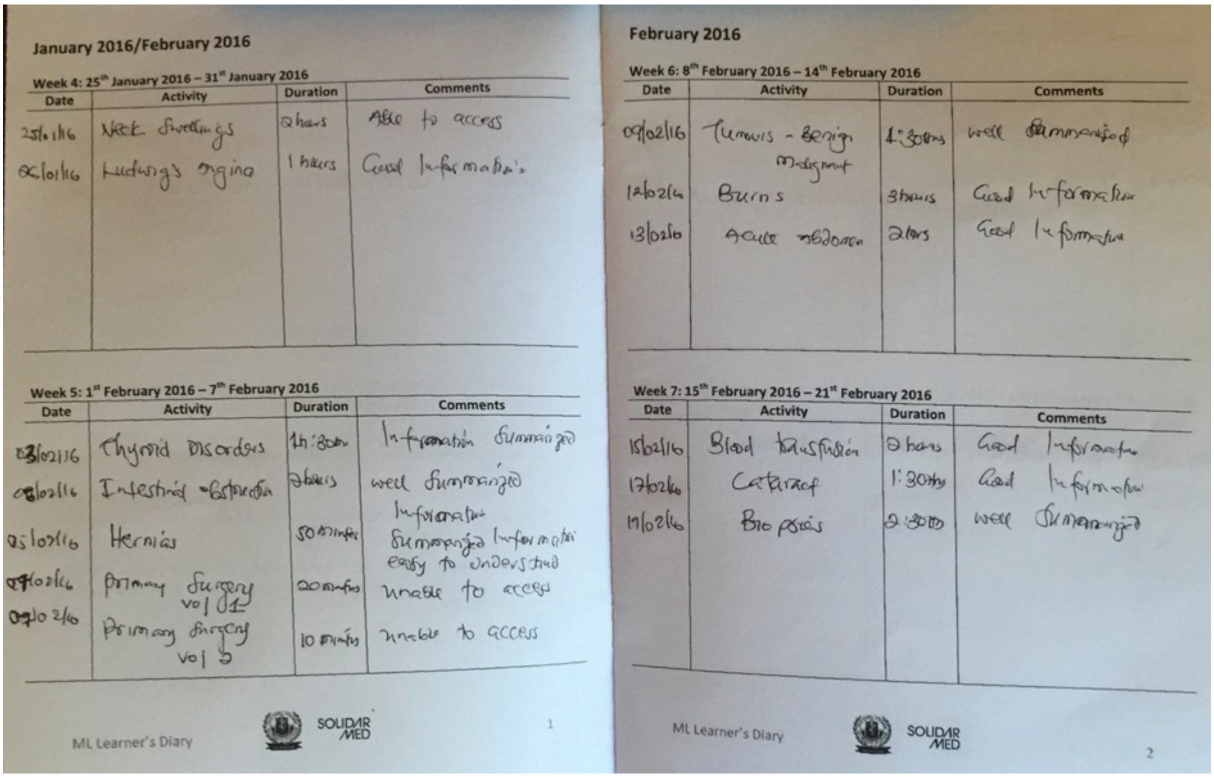

January 2016/February 2016

Week 4: 25th January 2016 – 31st January 2016

Date	Activity	Duration	Comments
25/01/16	Neck swellings	2 hours	Able to access
26/01/16	Ludwig's angina	1 hours	Good information

Week 5: 1st February 2016 – 7th February 2016

Date	Activity	Duration	Comments
03/02/16	Thyroid Disorders	1h:30m	Information summarized
03/02/16	Intestinal obstruction	3 hours	well summarized information
05/02/16	Hernias	50 minutes	Summarized information easy to understand
07/02/16	Primary Surgery vol 1	20 minutes	unable to access
07/02/16	Primary Surgery vol 2	10 minutes	unable to access

February 2016

Week 6: 8th February 2016 – 14th February 2016

Date	Activity	Duration	Comments
09/02/16	Tumours - Benign Malignant	1:30 hrs	well summarized
12/02/16	Burns	3 hours	Good information
13/02/16	Acute abdomen	2 hrs	Good information

Week 7: 15th February 2016 – 21st February 2016

Date	Activity	Duration	Comments
15/02/16	Blood transfusion	2 hours	Good information
17/02/16	Cataract	1:30 hrs	Good information
19/02/16	Biopsies	2:30 hrs	well summarized
